# Models of Impulsivity with a Focus on Waiting Impulsivity: Translational Potential for Neuropsychiatric Disorders

**DOI:** 10.1007/s40429-014-0036-5

**Published:** 2014-10-03

**Authors:** Valerie Voon

**Affiliations:** 1 Department of Psychiatry, University of Cambridge, Cambridge, CB2 0QQ UK; 2 Behavioural and Clinical Neuroscience Institute, University of Cambridge, Cambridge, UK; 3 Cambridgeshire and Peterborough NHS Foundation Trust, Cambridge, UK

**Keywords:** Waiting impulsivity, Premature responding, Impulsivity, Addiction, 5-CSRT

## Abstract

Waiting impulsivity, also known as premature or anticipatory responding, is well established in preclinical studies through the 5-Choice Serial Reaction Time (5-CSRT) task. Waiting impulsivity is important in disorders of addiction. Preclinical studies suggest a role both as a predictor, and as a consequence, in disorders of addiction. Here we discuss the relationship between the preclinical 5-CSRT and translational fidelity in newly developed translational tasks. Preclinical and clinical literature relevant to premature responding and disorders of addiction are reviewed. Understanding which processes are critical to premature responding is important in understanding the nature of premature responding. Premature responding may also have overlaps with motivational processes, proactive response inhibition, tonic inhibitory processes, and delay discounting.

## Introduction

Waiting impulsivity, also known as premature or anticipatory responding or impulsive action, is well characterized in preclinical rodent studies and more recently translated in human studies. Waiting impulsivity has potentially important clinical implications in disorders of addiction, as preclinical evidence suggests it can play a role both as a predictor, and as a consequence of disorders of addiction. Impulsivity is generally defined as the predisposition to act with a low or inadequate degree of deliberation, forethought, or control [1]. Waiting impulsivity is a subtype of impulsivity defined operationally as the tendency to respond before target onset, and can be assessed in rodents using the 5-Choice Serial Reaction Time Task (5-CSRTT) [2••]. The broader concept of impulsivity is a heterogeneous construct, which can be subdivided into: waiting impulsivity; motor response inhibition (the ability to cancel or restrain a pre-potent motor response); and decisional forms, including delay discounting (the tendency to select an immediate smaller reward over a larger delayed reward); or reflection impulsivity (the tendency towards rapid decisions, without adequate accumulation or consideration of the evidence). These subtypes have overlapping yet dissociable neural substrates [3]. Here we focus on evidence for preclinical and clinical evidence of waiting impulsivity, and its relationship to disorders of addiction. We first discuss the rodent 5-CSRTT and the role of state and trait effects in disorders of addiction. A brief summary of neural and neurochemical correlates from the preclinical literature is reviewed. This review focuses predominantly on newly developed translational tasks for humans including the 4-Choice Serial Reaction Time Task (4-CSRTT), which was developed to maintain translational fidelity with the 5-CSRTT. We then discuss cognitive processes that may contribute to further understanding the nature of human premature responding.

## Preclinical

### 5-Choice Serial Reaction Time Task

Here we describe the rodent 5-CSRTT for the purposes of comparison with the human task. In the preclinical 5CSRTT, rodents are placed in a box with five apertures and wait for a brief 0.5 second light cue or target predicting reward to appear behind one of the apertures before performing a nose-poke at the aperture. Accurate responding is rewarded with a food pellet collected at the food dispenser. Premature responding is measured as an early nose-poke before cue or target presentation and is punished by a five second time-out (house light extinguished and no delivery of food reward) (Robbins 2001). A failure to respond during the limited hold (a specified time interval after target onset and time for subject to respond), is recorded as an omission and punished by a five second time-out. Responses at a non-illuminated aperture are recorded as incorrect responses and are also punished by a five second time out. Repeated responding at the aperture is recorded as perseverative responding. The accuracy rate of target detection is calculated as the percentage of correct responses to the total number of correct and incorrect responses. Rodents are typically trained extensively on a fixed inter-trial interval (e.g., five second duration between food collection and light or target onset), with testing occurring on a prolonged inter-trial interval (e.g., seven seconds).

### State and Trait Effects

Premature responding has both trait (an endophenotypic predictor or risk factor for the development of a disorder), and state (a secondary consequence of exposure), effects dependent on the substance. Both stimulants and nicotine have preclinical evidence for both state and trait effects. High levels of premorbid premature responding in rodents predicts greater addiction-like behavior to cocaine defined operationally as: increased motivation to take cocaine; inability to inhibit drug seeking; and continued drug use despite aversive consequences [4•]. Greater premature responding for up to two weeks of abstinence, following chronic methamphetamine exposure, suggests premature responding can also be secondary to methamphetamine exposure [5]. In rodents, high premorbid premature responding predicts greater motivation to initiate and maintain nicotine use [6•], and nicotine increases premature responding [7].

Evidence for a role for premorbid impulsivity in predicting the development of alcohol use disorders is less clear. Premature responding is associated with alcohol-preferring mice strains as compared to non-alcohol preferring strains [8•], and is also associated with greater withdrawal severity from chronic alcohol amongst different mice strains [9]. Acute alcohol exposure [10], and early (but not late), abstinence following chronic alcohol exposure, are also associated with increased premature responding in rodents [11], suggesting that premature responding can be secondary to both acute and chronic alcohol exposure. High premorbid premature responding is also associated with greater escalation of sucrose-seeking behavior and reinstatement following extinction in rodents [12•]. The association between premature responding and strain differences is less clear, as premature responding in 15 different strains of mice is not associated with sucrose acquisition or preference [9].

### Neural Networks and Neurochemistry

In rodents, premature responding implicates a network including the nucleus accumbens, infralimbic cortex, and subthalamic nucleus. Dopaminergic, noradrenergic, serotonergic, and GABA-ergic mechanisms have also been implicated in premature responding. The evidence for a role for these neural regions and neurochemistry in premature responding is briefly reviewed.

Acute amphetamine increases premature responding in rodents, an effect attenuated by 6-hydroxydopamine lesions of the nucleus accumbens, and by D1/2 receptor antagonists [13]. High impulsive rodents have lower ventral striatal D2/3 receptor availability [14], and have lower left grey matter density in the nucleus accumbens core [15]. Atomoxetine, a selective norepinephrine-reuptake inhibitor, also dose-dependently decreases premature responding [16]. An opponency effect has been observed in the nucleus accumbens core and shell: microinfusion of methylphenidate, a mixed dopamine/norepinephrine reuptake inhibitor, in the core (but not the shell), is associated with increased premature responding [17]. In contrast, microinfusions of atomoxetine into the shell (but not the core), decreases premature responding. In rodents, infusion of quinpirole (a D2/3 agonist), into the nucleus accumbens core increases premature responding in high impulsive rodents, but within the shell increases locomotor activity [18]. High impulsive rodents also had decreased nucleus accumbens core glutamate decarboxylase (GAD 65/67), an enzyme implicated in the conversion of glutamate to GABA [15]. Furthermore, experimentally decreasing mRNA gene expression of GAD 65/67 increased impulsivity in low impulsive rodents.

Lesions of the infralimbic cortex (equivalent to the human subgenual anterior cingulate- SgACC), also increase premature responding [19]; although neither methylphenidate, nor atomoxetine, in the infralimbic cortex influence premature responding [17]. Central serotonin depletion and prefrontal 5-HT2C receptor antagonism is also associated with greater premature responding in rodents, whereas prefrontal 5-HT2A receptor antagonism is associated with a decrease in premature responding [20]. Similarly, 5HT2A receptor antagonists have been shown to decrease cocaine-induced premature responding in rodents [21], and 5HT2C receptor agonists have been shown to decrease nicotine-induced premature responding [22]. Lesions of the subthalamic nucleus in rodents are also associated with a robust enhancement in premature responding [23, 24].

## Clinical

### Premature Responding Tasks

There are several existing tasks assessing anticipatory, early or premature responding that have been tested in humans including the 4-CSRTT, Continuous Performance Task, Traffic Light task, Simon task and Sx-5CSRTT.

#### 4-Choice Serial Reaction Time Task (4-CSRT)

The 4-CSRT task was developed based on the rodent 5-CSRT. Subjects are seated in front of a touch screen [25••]. When four boxes appear on the screen, the subject presses and holds down the space bar on the keyboard with their dominant index finger indicating the ‘cue onset’ time. After a specified period (cue-target interval), a green circle target appears briefly and randomly in one of the four boxes. Subjects release the space bar and touch the box in which the target appeared. Baseline blocks without monetary feedback are used to individualize monetary feedback amounts for subsequent blocks, based on the individual’s mean fastest reaction time and standard deviation. The four Test blocks with monetary feedback were optimized, to increase premature responding, and varied by target duration and variability of the cue-target interval and the presence of distractors. Accurate and timely responses were followed by different reward magnitude outcomes, depending on the speed of responding. The primary outcome measure is the premature release of the space bar prior to target onset. Following a premature release, subjects were required to complete the trial by touching the screen, which was followed by a feedback slide indicating to ‘Keep going,’ without reward (equivalent to a time out penalty). Incorrect responses are errors of commission (wrong box touched after target onset). Other outcome measures included accuracy (correct responses/ correct + incorrect responses); late responses (late responses/correct fast responses + late responses, corresponding to errors of omission in the rodent paradigm); and total amount won.

Using the 4-CSRTT, our research group has shown that relative to healthy volunteers, premature responding is elevated in subjects abstinent from alcohol and methamphetamine use disorders, recreational cannabis users, and in current (but not ex- or non-), smokers [25••]. The observation in smokers suggests either a role for premature responding as a consequence of nicotine or that those with lower premature responding may be more effective at abstinence. There were no differences in premature responding observed in obese subjects, with or without, binge eating disorder. However, this may be related to testing in the context of satiety rather than food deprivation; use of a conditioned reinforcer (monetary outcome), rather than a primary reinforcer (food); or testing subjects with lower levels of obesity, rather than morbid obesity. Using this same task, we have also shown that premature responding in humans increases with tryptophan depletion [26•], or with a decrease in central serotonin levels.

We have further shown that premature responding in humans correlates negatively with increasing age, consistent with the trajectory of impulsivity with age [27]. Although we did not show a correlation between premature responding and impulsivity questionnaires in one study [25••], the tryptophan depletion study did show a correlation with impulsivity questionnaires [26•]. Premature responding did not correlate with IQ, depression scores, accuracy, reaction time, late responses, amount of money won, or motivation for monetary feedback [25••].

#### Continuous Performance Task

The original rodent 5-CSRTT was developed based on a human serial reaction time task or continuous performance task (CPT). A more demanding version of the CPT, the Immediate and Delayed Memory Task (IMT/DMT), has been used to measure impulsive action in a cross-species translational study [28, 29]. Rodents were tested on the 5-CSRTT with both amphetamine and atomoxetine manipulation, and humans were tested on the IMT/DMT. The IMT/DMT consists of randomly generated five-digit numbers. In the IMT, subjects respond to the target stimuli when two identical randomly generated five-digit numbers are presented in sequence. In the DMT, a distracter stimulus (e.g., 12345), appears three times between the target stimuli and should be ignored. Impulsive action errors were measured as the ratio of commission errors (responses to non-identical numbers), to correct detections; or the equivalent action restraint errors in go/no go tasks. Although this task focuses on commission errors, as discussed below in the section on processes related to waiting impulsivity, the role of response inhibition prior to motor initiation may be of relevance in premature responding.

#### Traffic Light Task

In the Traffic Light task, subjects respond to optimize time-sensitive risky rewarding outcomes [30, 31•]. Subjects view a red light that turns amber and then green, and must respond rapidly to the green Go signal to obtain a reward. The reward value is greatest at the minimum possible latency following the Go signal, and declines steeply with increasing reaction time after Go signal onset. The amber duration is variable so the time of onset of the Go signal cannot be predicted. To optimize the reward, rather than waiting for the Go signal and responding reactively, subjects should respond during the anticipatory amber period prior to Go signal onset. Such a response has an element of a risk estimation and choice, since the onset of the Go signal cannot be accurately predicted: a successful motor response immediately after the Go signal is optimally rewarded, but responding prior to the Go signal is penalized with a small fixed penalty. The outcome measure is the frequency of penalized trials and the overall reward obtained. Anticipatory responding, measured using this task, correlated with the lack of premeditation on the UPPS Impulsive Behaviour Scale, and was negatively correlated with age in healthy volunteers [30]. The anticipatory response in the Traffic Light task thus occurs in the context of a risky choice, response selection, sensitivity to reward and loss, and capacity for time estimation.

#### Simon Task

Anticipatory responding has also been reported using the Simon task, in which an irrelevant stimulus evokes an early strong response impulse that interferes with goal-directed action; with subsequent response inhibition affecting later impulsive actions [32••, 33, 34]. In the Simon task, subjects view a central fixation point followed by a blue or green circle appearing to the left or right of the fixation point. Subjects respond with either the left or right finger, dependent on an indicated colour response mapping. In corresponding trials the spatial location of the circle matches with the colour response mapping (e.g., blue circle mapped to right finger appears on right). In non-corresponding trials, the spatial location of the circle was opposite that of the colour response mapping (e.g., blue circle mapped to right finger appears on left). The analysis is based on an activation-suppression model in which accuracy is parcellated into reaction time bins; thus differentiating early stimulus-driven, prepotent, impulsive, but erroneous actions; and subsequent later selective suppression of these impulsive actions, to facilitate selection of the correct action. In this task, the early erroneous motor response occurs *after* the stimulus onset and does not specifically address anticipatory or waiting impulsivity. The impulsive response is driven by the irrelevant stimulus capturing a prepotent response, which can either facilitate or interfere with the actual goal-oriented response. The task thus assesses prepotent stimulus-induced impulsive responding in the context of conflict, and dissociates this measure from the influence of response suppression and response selection.

This early impulsive response in healthy volunteers is associated with activity in the pre-supplementary motor area (pre-SMA), and the late response with right inferior frontal cortex activity [34]. Impairment of the pre-SMA using transcranial magnetic stimulation in the Simon task decreased impulsive responses, particularly when subjects were being explicitly rewarded for fast accurate responses suggesting an influence of motivation or reward anticipation [35]. The authors suggest a parallel influence of prefrontal regions, since pre-SMA impairment was associated with greater connectivity of the inferior frontal gyrus and subthalamic nucleus, along with decreased impulsivity. Subthalamic deep brain stimulation in Parkinson’s disease patients has been shown to enhance early stimulus-driven impulsive responding in the Simon task, but to improve selective inhibition of late responses [32••]. Parkinson’s patients with medication-induced impulse control behaviors also had less early impulsive responding, relative to those without impulse control behaviors [36], suggesting this form of impulsivity is not relevant to the disorder.

#### Sussex-5-Choice Serial Reaction Time Task

In the Sussex-5-Choice Serial Reaction Time task (Sx-5CRST), subjects respond to a highlighted stimulus from one of five moving stimuli using their dominant hand [8•]. Premature responding is measured as a response prior to onset of the highlighted stimulus, and penalized by a five second time out. Both fixed and variable interval conditions are tested along with both conditions under a dual-task condition, with responding with a key press to a tone with the non-dominant hand. Subjects are not explicitly rewarded for correct responding. Thus, the task may focus more on motoric impulsivity elements, rather than on impulsivity in the context of reward anticipation.

Using this task, male binge drinking subjects had higher premature responding, compared to healthy males in the fixed interval condition [8•]. In the distraction condition, binge drinkers of both genders were associated with greater premature responding, along with a greater number of errors relative to healthy volunteers, suggesting a role for attentional load or task difficulty in mediating these findings.

#### Summary

Several tasks have been developed to assess waiting impulsivity in human subjects, some of which may be influenced by other features including response inhibition, anticipatory responding in the context of risk, stimulus-induced prepotent responses, and the role of reward anticipation.

### Processes that Might Influence or are Related to Waiting Impulsivity

Waiting impulsivity as measured using the rodent 5-CSRT assesses premature responding in the context of anticipation of cues predicting reward. There are several cognitive processes that may play a role in premature responding which include attention, motivation, delay discounting, response inhibition, sensitivity to reward, and to negative outcomes.

#### Attention

The accuracy measure in the 4-CSRT accounts in part for the non-specific influences such as attentional capacity, motivation, or motor behavior, since both correct and incorrect responses require the same motor effort [2••]. We did not show any correlation between accuracy measures and premature responding, suggesting non-specific attention can be dissociated from premature responding [25••]. Under tryptophan depletion, we show enhanced premature responding in humans as tested using the 4-CSRT, along with enhanced accuracy, suggesting that attentional deficits are unlikely to account for the increase in premature responding [26•].

#### Sensitivity to Negative Outcomes

Both the 4-CSRT and Sx-5CSRT have time out penalties following a premature response. During task development of the 4-CSRT, the introduction of an explicit monetary loss feedback to the premature response resulted in a marked decrement in premature responding in healthy volunteers. Sensitivity to negative feedback may be relevant in disorders of addiction, as rodent models of binge drinking and human binge drinking subjects have shown decreased sensitivity to aversive reinforcement [37]. Our group has also shown that young-adult BD subjects are more risk-seeking when anticipating loss outcomes, similarly suggesting impaired sensitivity to aversive outcomes [38].

#### Motivational Processes

One possible relevant mechanism is that of a generalized enhancement in incentive motivational responding. The 4-CSRT has, embedded within the design, a means of assessing reaction time; either as an index of motivational responding, or reward sensitivity. Subjects first learn to respond as quickly as possible to the target in Baseline 1, used to individualize reward feedback. Following Baseline 1, Test Block 1 has a similar design as Baseline 1, although responding to the target is now followed by monetary feedback. A second baseline block, Baseline 2 (equivalent to Baseline 1), follows Test Block 1. Thus, Baseline 2 follows instrumental responding to a target with monetary feedback, consistent with testing in extinction without feedback. The Motivation Index, or difference between the baseline blocks, might thus allow the assessment of a change in reaction time, as an index of motivation or reward sensitivity; testing either differences in responding to over-learned instrumental goal-directed behaviors tested in extinction, or if the target becomes conditioned to the reward, then testing responding to the conditioned stimulus in extinction.

Using the 4-CSRT we show that Motivation Index, which was unrelated to premature responding, is decreased in abstinent alcohol-dependent subjects, and is negatively correlated with both the severity of alcohol dependence and binge eating [25••].

#### Delay Discounting

In the rodent literature, premature responding correlates with delay discounting [39], suggesting overlaps in the impairment of ‘waiting’ for reward. However, in the human 4-CSRT, delay discounting as tested using the hypothetical Monetary Choice Questionnaire was not correlated with premature responding [25••]. There are several reasons we might observe such a dissociation.

The neural substrates of premature responding and delay discounting overlap particularly in the ventral striatum, but are not identical. Lesions of the rodent nucleus accumbens core, amygdala, and hippocampus increase delay discounting, suggesting that these structures are critical to the evaluative process [40–42]. In human fMRI studies, the ventral striatum, dorsolateral prefrontal cortex, insula, amygdala, posterior cingulate, and parietal cortex have been implicated in delay discounting for secondary [43–46], and primary rewards [47]. The orbitofrontal cortex is also implicated in time-discounting of rewards [48], with lesions of the rodent medial and lateral orbitofrontal cortex associated with a decrease and increase in delay discounting respectively [49]; and orbitofrontal cortex lesions in humans is associated with greater delay discounting [50].

Delay discounting in rodents is typically tested by determining the indifference point between an immediate choice of a small reward and a delayed choice of a larger reward, by manipulating the duration of the delay or the reward magnitude. Rodent studies use reward feedback in real-time over short delays (e.g., in seconds), whereas the Monetary Choice Questionnaire in humans uses hypothetical monetary feedback over long delays (e.g., in days equivalent to weeks and months). Discounting tasks in real time with feedback such as the Experiential Discounting Task have been developed for human studies, and might show a different relationship with premature responding [51]. The short duration for the delayed reward has also been shown in human studies to differ from longer delays by being better modeled by an exponential, rather than a hypothetical, discount function [52]. What aspect of delay discounting might be related to premature responding is also relevant, as delay discounting itself can be parcellated into several cognitive mechanisms, including (but not limited to), the incentive salience of the immediate choice, the discounting effects of the delayed reward, delay aversion, the uncertainty of the delayed choice, magnitude effects, time estimation or decreasing subjective valuation of increasing magnitude.

#### Response Inhibition

Response inhibition may be implicated in premature responding, particularly in the context of tonic inhibitory mechanisms and proactive stopping or stopping prior to initiation of a motor action. The stop signal task assesses action cancellation of a prepotent motor action that has already started, whereas the Go/NoGo task assesses action restraint of a prepotent motor action that is not yet initiated, the latter of which may be more relevant. In the rodent 5CSRTT, differences between premature responding and false errors (analogous to Go/NoGo commission errors), have been highlighted [53]. In rodent and human studies, there is no relationship between premature responding and the stop signal task [25••], but the relationship with the more relevant role of action restraint in humans has not yet been tested. The neural substrates of the stop signal and Go/NoGo tasks overlap and implicate the pre-SMA, right inferior frontal gyrus, caudate, and subthalamic nucleus (STN) [54–56]. These neural regions overlap with premature responding particularly in the STN.

Proactive stopping [57], or preparing to suppress a response tendency rather than reactive stopping after signal onset, may be relevant to premature responding. The premature responding task does not include external stop signals. When stop signals are expected and introduced in a task, behavioral responses change with proactive response strategies implemented, thus increasing response thresholds, slowing the go reaction time, and increasing the likelihood of stopping [57, 58]. Proactive stopping with internal, rather than external, signals may also be relevant in premature responding. The STN is hypothesized to be engaged in influencing response suppression by inhibition of thalamo-cortical pathways via both reactive and proactive models. In reactive stopping, the STN is proposed to relay a reactive global No-Go signal to inhibit execution during high conflict tasks, thus allowing greater time to integrate information [59, 60]. Alternatively, in the proactive inhibition model of the STN, inhibitory processes are suggested to generalize in which the default state is a tonic inhibitory process involved in the suppression of automatic or prepotent responses. This proactive stopping and tonic inhibitory process may be relevant to premature responding in the context of prepotent reward anticipation.

## Conclusion

Waiting impulsivity is an important process in disorders of addiction. Preclinical studies suggest a role both as a predictor and as a consequence in disorders of addiction. We suggest that one of the human analogues, the 4-CSRT, has strong translational fidelity with the rodent version of the 5-CSRT task. Which processes are the elements crucial to premature responding is important in understanding the nature of premature responding. These include impulsivity, in the context of the anticipation of a cue predicting reward, the role of non-specific anticipatory motor responding, responding in the context of an irrelevant stimulus inducing a prepotent response, or in the context of risk. The relationship between premature responding and motivational processes, proactive response inhibition, tonic inhibitory processes, and delay discounting remain to be further studied. An in depth understanding of the nature of premature responding in humans allows us to develop more specific targeted behavioral measures and interventions.
